# Reply to Zheng et al.: Clinical metabolomics: Detailed analysis by nontargeted method is complementary to large-scale studies

**DOI:** 10.1073/pnas.2120693119

**Published:** 2022-01-24

**Authors:** Hiroshi Kondoh, Takayuki Teruya, Yung-Ju Chen, Yasuhide Fukuji, Mitsuhiro Yanagida

**Affiliations:** ^a^Geriatric Unit, Graduate School of Medicine, Kyoto University, Kyoto 606-8507, Japan;; ^b^G0 Cell Unit, Okinawa Institute of Science and Technology Graduate University, Okinawa 904-0495, Japan;; ^c^National Hospital Organization Ryukyu Hospital, Okinawa 904-1201, Japan

Accurate, nontargeted, comprehensive analysis of metabolites provides essential information about cutting-edge clinical questions (1). Zheng et al. (2) question the legitimacy of our recent discovery of dementia markers using whole-blood metabolomics (3).

First, they express concern about the sample size of our study (*n* = 16) (2). However, our findings (3) do not conflict with other studies. Rather, they are well supported by other work. In addition to our study, a recent large-scale study of metabolomics (*n* = 496) identified ergothioneine (ET) as a dementia marker (4), while frail elderly patients, manifesting cognitive impairment, also display a decline in ET levels (5). Moreover, the statistical analysis in our study was validated by a reviewer for this journal with expertise in statistics, during the review process. Thus, whole-blood metabolomics is a useful approach, since some dementia markers like ET are abundant in red blood cells.

Second, Zheng et al. (2) note the age difference between test subjects with dementia and those who served as controls in our study (3). It is possible that aging increases the risk of neurodegenerative diseases (6). Previously, we reported individual differences in 126 blood metabolites between young (29 ± 4 y) and elderly people (81 ± 7 y) (7). While 14 blood metabolites were listed as aging markers, most of the dementia markers, including ET, were not included in this list (3). Indeed, an age-matched study for dementia markers drew conclusions similar to ours (4).

Third, we agree that environmental factors, such as food and drugs, may affect metabolomic profiles. Patients with mild cognitive impairment (MCI) may progress to Alzheimer’s disease (6), but, generally, MCI does not require drug administration. Metabolomics also identified ET as a marker for MCI (5, 8), implying that drugs for dementia, like memantine, do not affect our conclusions. Accumulating data suggest that caffeine has a protective role on cognitive function through its stimulation of the central nervous system, consistent with our findings (9, 10).

Usually, large-scale epidemiological surveys target specific metabolites, while our whole-blood metabolomics provide detailed, nontargeted, comprehensive analysis (3). Thus, findings from the two approaches complement each other, which is why our nontargeted approach constitutes a significant, incremental advance in the field of disease metabolomics.
